# Open lunate enucleation

**DOI:** 10.11604/pamj.2015.21.289.6678

**Published:** 2015-08-20

**Authors:** Monsef El Abdi, Adil Lamkhanter

**Affiliations:** 1 Department of Orthopaedic Surgery «1», Military Hospital of Instruction Mohammed V, Rabat, Morocco

**Keywords:** Lunate, enucleation, dislocation

## Image in medicine

A 32 year old man, right-handed soldier, was admitted to Emergency Department two hours after undergoing right wrist injury following a 2 meter fall onto the hand, in hyperextension. Clinical examination revealed a deformity of the wrist with an obvious open lunate enucleation (A). The neurovascular status was intact. Standard radiograph of the wrist demonstrated a completely enucleated lunate, associated with scaphoid and radial styloid fracture (B). Computed tomography (CT) scan confirmed diagnosis of trans-radial styloid, trans-scaphoid, perilunate dislocation (C and D). The lunate enucleation was treated by open reduction through a volar approach and internal fixation of associated injuries. Wrist reduction was maintained using K-wires placed through the scapholunate and scaphocapitate. The material was removed after 3 months. The control during one year postoperatively, there was no evidence of complications. The patient had a comfortable range of motion in his right wrist.

**Figure 1:**
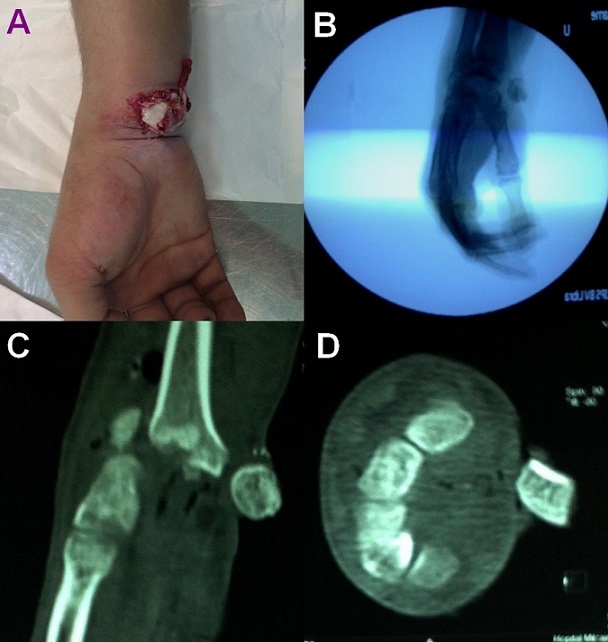
(A) clinical aspect at admission revealing an open enucleation of a carpal bone, (B) X-ray of the left wrist shows a lunate dislocation, (C and D) computed tomography scan (transverse and sagittal cut) shows lunate enucleation with scaphoid and radial styloid fracture

